# Development of Iron Sequester Antioxidant Quercetin@ZnO Nanoparticles with Photoprotective Effects on UVA-Irradiated HaCaT Cells

**DOI:** 10.1155/2021/6072631

**Published:** 2021-08-25

**Authors:** Muhammad Farrukh Nisar, Maryam Yousaf, Muhammad Saleem, Hamad Khalid, Kamal Niaz, Mustansara Yaqub, Muhammad Yasir Waqas, Arsalan Ahmed, Muhammad Abaid-Ullah, Jinyin Chen, Chuying Chen, Kannan R. R. Rengasamy, Chunpeng (Craig) Wan

**Affiliations:** ^1^Jiangxi Key Laboratory for Post-Harvest Technology and Nondestructive Testing of Fruits & Vegetables, Collaborative Innovation Center of Post-Harvest Key Technology and Quality Safety of Fruits and Vegetables in Jiangxi Province, College of Agronomy, Jiangxi Agricultural University, Nanchang 330045, China; ^2^Department of Physiology and Biochemistry, Cholistan University of Veterinary and Animal Sciences (CUVAS), Bahawalpur, Punjab 63100, Pakistan; ^3^Key Laboratory of Crop Physiology, Ecology and Genetic Breeding, Ministry of Education, Jiangxi Agricultural University, Nanchang, 330045 Jiangxi, China; ^4^Interdisciplinary Research Centre in Biomedical Materials (IRCBM), COMSATS University Islamabad, Lahore Campus, Lahore 54000, Pakistan; ^5^Department of Chemistry, Faculty of Sciences, University of Kotli, Azad Jammu and Kashmir, Pakistan; ^6^Institute of Advance Study, Shenzhen University, Nanshan District, Shenzhen, Guangdong 518060, China; ^7^Department of Pharmacology and Toxicology, CUVAS, Bahawalpur, Punjab 63100, Pakistan; ^8^Department of Life Sciences, Khwaja Fareed University of Engineering & Information Technology, Rahim Yar Khan, Pakistan; ^9^College of Materials and Chemical Engineering, Pingxiang University, Pingxiang 337055, China; ^10^Green Biotechnologies Research Centre of Excellence, University of Limpopo, Private Bag X1106, Polokwane Sovenga 0727, South Africa

## Abstract

**Background:**

Solar ultraviolet radiation A (UVA, 320-400 nm) is a significant risk factor leading to various human skin conditions such as premature aging or photoaging. This condition is enhanced by UVA-mediated iron release from cellular iron proteins affecting huge populations across the globe.

**Purpose:**

Quercetin-loaded zinc oxide nanoparticles (quercetin@ZnO NPs) were prepared to examine its cellular iron sequestration ability to prevent the production of reactive oxygen species (ROS) and inflammatory responses in HaCaT cells.

**Methods:**

Quercetin@ZnO NPs were synthesized through a homogenous precipitation method, and the functional groups were characterized by Fourier transform infrared (FTIR) spectroscopy, whereas scanning electron microscopy (SEM) described the morphologies of NPs. MTT and qRT-PCR assays were used to examine cell viability and the expression levels of various inflammatory cytokines. The cyclic voltammetry (CV) was employed to evaluate the redox potential of quercetin-Fe^3+^/quercetin-Fe^2+^ complexes.

**Results:**

The material characterization results supported the loading of quercetin molecules on ZnO NPs. The CV and redox potential assays gave Fe-binding capability of quercetin at 0.15 mM and 0.3 mM of Fe(NO_3_)_3_. Cytotoxicity assays using quercetin@ZnO NPs with human HaCaT cells showed no cytotoxic effects and help regain cell viability loss following UVA (150 kJ/m^2^).

**Conclusion:**

Quercetin@ZnO NPs showed that efficient quercetin release action is UV-controlled, and the released quercetin molecules have excellent antioxidant, anti-inflammatory, and iron sequestration potential. Quercetin@ZnO NPs have superior biocompatibility to provide UVA protection and medication at once for antiphotoaging therapeutics.

## 1. Introduction

Iron plays a major role in various biological processes of the body and is stored in different proteins and/or as free iron in the cell [1]. Huge amounts of iron are scattered throughout the cytosol and in various cellular organelles especially mitochondria, nucleus, and lysosomes [2, 3]. The free iron or cellular labile iron pool (LIP) is comprised of redox-active Fe^2+^ that can potentially damage cell signalling at multiple sites by forming reactive oxygen species (ROS). The iron-generated ROS can damage cellular lipid bilayers, genetic material (DNA and RNAs), and proteins [4]. Many of the extrinsic factors such as ultraviolet radiation A (UVA) and B (UVB), along with environmental Benz(e)acephenanthrylene (BeA) and chrysene, can increase the oxidative stress, damage DNA, and apoptosis in human skin cells [5, 6]. Similarly, intrinsic factors such as free iron in spite of the essential roles in the body can be toxic if it accumulated in excess following UVA exposure which may lead to various diseases including cancer, ischemia-reperfusion injury in transplantations, and aging-associated neurodegenerative issues [7]. Chronic ROS efflux creates the cellular oxidative stress in human skin. The oxidative stress leads to skin cancer [8–10] and damages the natural defence system of the skin [9, 11, 12]. ROS adversely deteriorates biomembranes and biomolecules and induces the expression of matrix metalloproteinases (MMPs) [13], which produce damaged or photoaged skin [14].

Plant active compounds efficiently quench these free ROS and neutralize them to reduce cellular components being damaged and reduce disease progression or incident. On the other hand, many phytochemicals directly quenched the free cellular iron (Fe^2+^) and reduce iron-mediated cellular toxicity and help maintain cellular redox homeostasis [15].

Various environmental factors such as ultraviolet radiations (UVRs), heat, smoke, and chemicals contribute to cellular ROS efflux that triggers the expression of inflammations and induces aging or photoaging and carcinogenesis [12, 14, 16]. UVA radiation is the highest (95%) of all the UVs reaching the biosphere, and it is a significant risk factor for the above-mentioned conditions, polymorphous light eruption (PLE), and sunburns [17].

Quercetin is a well-known dietary flavonoid present in vegetables, fruits, tea, and brewed drinks [18]. Quercetin has potential beneficial effects on disease prevention through antioxidant properties by scavenging ROS [19] and even antitumor activities [20, 21]. Quercetin has been reported to be cytoprotective against various ailments and a potent antioxidant [18]. However, hydrophobicity, strong lipophilic nature, and lower bioavailability hinder the achievement of localized treatments using quercetin.

Zinc oxide (ZnO) is a cheap and ingenious metal oxide used in skincare products for UVR shielding and as a drug carrier [22]. Moreover, it is well known that ZnO nanoparticles (NPs) could transform between the hydrophobic to hydrophilic states following UVR exposure and the dark period to unload the drugs [22, 23]. The current study was aimed at utilizing low doses of UVA light (320-400 or 365 nm) along with ZnO-loaded quercetin (quercetin@ZnO) molecules to release the drugs. Following UVA exposures, quercetin@ZnO molecules release the drug for simultaneous skin protection and sunblocking by delivering maximum quercetin molecules to the targeted site. Moreover, the quenching of the free iron molecules, redox potential, anti-inflammatory effects of quercetin on the UVA-driven cytokines, and ROS burst in the HaCaT cells shall also be examined. The method applying quercetin@ZnO may be a useful therapeutic against UVA-related skin condition such as premature photoaging and requires further in-depth investigations for more clinical applications of this cotreatment (quercetin@ZnO+UVA) therapy.

## 2. Materials and Methods

### 2.1. Development of ZnO Nanoparticles (NPs) and Quercetin Encapsulation

The development of ZnO NPs was done following a previously described method [22]. Quercetin (CAS # 117-39-5) was purchased from Biopurify Phytochemicals Ltd., China (http://www.biopurify.com), and a small amount of quercetin (0.20 mg) was mixed and homogenized in 5 *μ*L of acetone and diluted into various mass ratios of quercetin to ZnO NPs (quercetin : ZnO; 1 : 100, 1 : 50, 1 : 10, 1 : 1, 10 : 1, 50 : 1, and 100 : 1). Deionized water (1 mL) was added before ultrasonication in high sound waves to evaporate acetone for a total of an hour. The excessive deionized water was removed through centrifugation (12000 rpm) for 10 min to obtain pure quercetin@ZnO NPs pelleted in the bottom, further dried using N_2_. Now, acetone (1 mL) was added and dissolved the loaded quercetin@ZnO NPs and was again centrifuged (12000 rpm) for a further 10 min for sharp separation. The various concentrations of quercetin@ZnO NPs in the acetone were examined using a UV spectrophotometer (Lambda 900, PerkinElmer, USA) in the wavelength range of 200-900 nm, which calculated the adsorption rate (AR) and drug loading capacity (LC) of quercetin on ZnO NPs in accordance with the standard curve of quercetin in acetone using the following equation

(1) $$\begin{array}{c} \text{AR}\%=\frac{W_{\text{in}}}{W_{\text{raw}}}\times 100\%,\\ \text{LC}\%=\frac{W_{\text{in}}}{W_{\text{ZnO}}}\times 100\%, \end{array}$$

where *W*_in_ is the weight of quercetin in acetone, *W*_raw_ is the raw weight of loading quercetin, and *W*_ZnO_ is the raw weight of ZnO for loading quercetin. The verification of results was done in three independent tests.

### 2.2. Characterization of ZnO NPs and Quercetin Release Studies

The shape and size of ZnO NPs were studied by scanning electron microscopy (SEM) (SU-70 HITACHI, Japan). The size distribution of NPs was observed at 25°C by using dynamic light scattering equipment (DLS, Zetasizer Nano S90, Malvern, UK) [22]. The element composition was examined through energy-dispersive spectroscopy (EDS) (X-MaxN, Oxford Instruments, UK). The infrared spectrum of ZnO NPs and quercetin@ZnO NPs was determined using a Fourier transform infrared spectrometer (FTIR) (Spectrum GX, PerkinElmer, USA).

The quercetin@ZnO NPs were selected having optimal AR value and used for UVA-controlled specific release studies. Then, we scattered the quercetin@ZnO NPs randomly on the glass slides exposed to a UVA light (365 nm, 12Watt) emitter at 37°C under dark conditions. A total of 5 experiment groups with triplicate were performed. Then, irradiate the quercetin@ZnO NPs with UV light, and at different times (1, 2, 4, 6, and 8 h), each group of quercetin@ZnO was poured off and cleaned using acetone (1 mL). The discharged quercetin concentration in the acetone from quercetin@ZnO NPs was examined using UV spectrophotometry which depicts its release or unloading behaviour. Meanwhile, quercetin@ZnO NPs with no UV irradiation were taken as controls.

### 2.3. Cell Culture Conditions and Pretreatment of Cells with Quercetin

Human epidermal keratinocytes (HaCaT) were kindly donated by Professor Julia Li Zhong, Photobiology Laboratory, College of Bioengineering, Chongqing University, China. HaCaT cells were maintained in Dulbecco's modified Eagle's medium (DMEM) culture medium at the cell culture lab, Interdisciplinary Research Center in Biomedical Materials (IRCBM), CUI, Lahore Campus, Pakistan. HaCaT cells were maintained in high-glucose DMEM (Gibco), having 10% (*v*/*v*) heat-inactivated fetal bovine serum (FBS) media and also containing 50 U/mL penicillin/streptomycin. Cells were kept under a humidified cell incubator chamber set at 37°C adjusted with 5% CO_2_. Cells were pretreated with a range of quercetin concentrations (0, 1.0, 2.5, 5.0, 10.0, and 20.0 *μ*M) found through cell viability under various drug doses. After finding the optimum concentration (20 *μ*M), cells were exposed to quercetin for 12 h and exposed to UVA, and then, stress-related studies were conducted.

### 2.4. UVA Source and Dosimetry

For the UVA treatment to the HaCaT cells, the UVA lamp with a specific waveband of 365 nm was used. The dosage was calculated by using standard measurement procedures provided with the lamp. An optimum dose range of UVA was set by applying a range of doses (0, 25, 75, 150, 300, and 450 kJ/m^2^) where the cell viability was about 75-80%.

### 2.5. Binding Assay of Ferric Ion (Fe^3+^) with Quercetin

A stoichiometry amount of Fe^3+^-binding quercetin molecules was measured with a spectrophotometer. An increased absorbance was observed due to the complex formation of Fe^3+^ with quercetin. Samples containing 0.3 mM quercetin, 100 mM KNO_3_, and 0.5 mM FeNO_3_ in 50 mM (4-(2-hydroxyethyl)-1-piperazine ethane sulphonic acid solution were prepared. Stability constant (log *k*) for quercetin/Fe^3+^ is measured by a ligand exchange method. After the addition of EDTA (chelating agent), the decrease in absorbance of Fe^3+^ by quercetin was observed. Each measurement was taken at 25°C using a spectrophotometer.

### 2.6. Measurement of Redox Potential by Cyclic Voltammetry (CV)

CV was employed to evaluate the redox potential of quercetin-Fe^3+^/quercetin-Fe^2+^ complexes. Voltammograms were recorded by using the PalmSens electrochemical sensor interface workstation. A three-electrode system was used comprising of glassy carbon as the working electrode (3 mm in diameter), saturated calomel electrode as the reference electrode, and platinum wire as the counter electrode. For electrochemical measurements, a solution of quercetin (4.0 mM) and Fe(NO_3_)_3_ (2.0 mM) in a 100 mM phosphate buffer solution with pH 7.0 was prepared and purged with nitrogen for about 20 min to remove any atmospheric oxygen before the experiment.

### 2.7. Cytotoxicity of Quercetin@ZnO NPs through the MTT Assay

HaCaT cells were placed at a density of 6 × 10^3^ in every well of a 96-well plate for 24 h and then pretreated with the quercetin for a further 12 h. Following pretreatment with quercetin, HaCaT cells were washed three times using sterilized phosphate buffer saline (PBS), and then, let them be incubated with DMEM having MTT (MTS, 3582, Promega) for about 2-4 h following the instructions of the manufacturer. Cell viability was examined with a microplate reader (Model 680, Bio-Rad, Hercules, CA, USA) adjusted at 490 nm. For quercetin@ZnO NPs, a similar procedure was adopted; i.e., HaCaT cells were seeded after 24 h and pretreated with various concentrations of ZnO, indole, quercetin, quercetin@ZnO, and ZnO/indole for an additional 12 h, followed by UVA irradiation (150 kJ/m^2^). The HaCaT cells were kept for recovery from harmful effects of UVA radiations for about 12 h in DMEM containing 1% (*v*/*v*) FBS. After 24 h post-UVA irradiation, the DMEM (10% FBS) will be changed with fresh DMEM (0.5% FBS) mixed with MTT and allowed to incubate for a further 2 h following instructions by the manufacturer. A microplate reader gave measurements about the viable HaCaT cells at 490 nm. The cytotoxic effect and viable cells under various combinations, i.e., ZnO, indole, quercetin, quercetin@ZnO, and ZnO/indole, were seen, and the percent of viable cells to that of control was taken.

### 2.8. Measurement of Intracellular ROS

In this study, HaCaT cells (2.5 × 10^5^) were seeded in 24-well plates and set for incubation for about 24 h; the cells were exposed to UVA (150 kJ/m^2^) as the UVA-treated group and pretreated with various concentrations of quercetin (0, 1.0, 2.5, 5.0, 10.0, and 20.0 *μ*M) to find an optimum concentration for later treatments. These pretreated cells (12 h) were also exposed to the UVA dose (150 kJ/m^2^) to check the level of intracellular ROS production in pretreated cells using the 2′,7′-dichlorofluorescein diacetate (DCFH-DA) Reactive Oxygen Species Assay Kit. Cells were then washed twice with sterilized PBS, and add 10 *μ*mol DCFH-DA (DCFH − DA : DMEM = 1 : 1000) (10 mM assay kit, Beyotime Inc., S-0033) into each well and incubate them at 37°C to detect cellular ROS levels. After 20 min of incubation following the manufacturer's procedure (Beyotime Inc., Shanghai, China), cells were given a wash three times with sterilized PBS. The HaCaT cells were examined, and photographs were taken using a fluorescent microscope (IX-71, Olympus Corp., Tokyo, Japan).

### 2.9. RNA Extraction, cDNA Synthesis, and Gene Expression Analysis (qRT-PCR)

Total cellular RNA was extracted from sham or treated (UVA/quercetin) cells using the TRIzol reagent (Invitrogen, Carlsbad, CA, USA) according to the instructions provided by the manufacturer and then digested with RNase-free DNase-I (Promega, Madison, WI, USA) to remove genomic DNA. The extracted RNA's purity was quantified as a ratio of OD260/OD280 and measured with a NanoDrop 1000 spectrophotometer (Thermo Scientific, New York, United States), and RNAs were stored at -80°C for later use. The cDNA was prepared from 1 *μ*g of the RNA by reverse transcriptase polymerase chain reaction (RT-PCR). According to the manufacturer's instructions, the RNA was reverse transcribed using SuperScript II Reverse Transcriptase and Random Primer Mix (Invitrogen, USA) in 25 *μ*L volume.

Total RNA content in cells was isolated from both groups using an RNA extraction kit (BioTeke Corporation, Beijing, China). First-strand cDNA was synthesized using PrimeScript® RT Reagent Kit with gDNA Eraser (Takara, Dalian, China). PCR amplification of the cDNA products (1 *μ*L) was performed with the PCR premix (BioTeke, Wuxi, China) and by using the following primer pairs (Table 1). Using a LightCycler apparatus (C1000 Touch, Bio-Rad, Hercules, CA, USA) with the Promega GoTaq® qPCR Master Mix (A6001) for qRT-PCR experiments, gene-specific primers (Table 1) will be used to analyze the expression levels of the gene. The qRT-PCR was performed in a StepOnePlus Real-Time PCR System using the SYBRVR Premix Ex Taq™ kit (Takara, Dalian, China). The qRT-PCR procedure included a series of cycles of 95°C for 10 s followed by 40 cycles of 95°C for 15 s, 60°C for 15 s, and 72°C for 30 s. Every experiment was laid in triplicate to analyze the relative expression level of the specific inflammatory genes. The GAPDH gene fragment, amplified with primers (Table 1), was used as an internal control.

### 2.10. Statistical Analysis

The data taken for three separate experiments in triplicate for each sample is represented as mean ± standard deviation (SD). The statistical significance was found by using Student's *t-*test, and *p* values of <0.05 for all relative experiments are set as significant.

## 3. Results

### 3.1. Development and Characterization of ZnO and Quercetin@ZnO NPs

#### 3.1.1. SEM Analysis of Quercetin-Loaded ZnO Nanoparticles (Quercetin@ZnO NPs)

**Figure 1:**
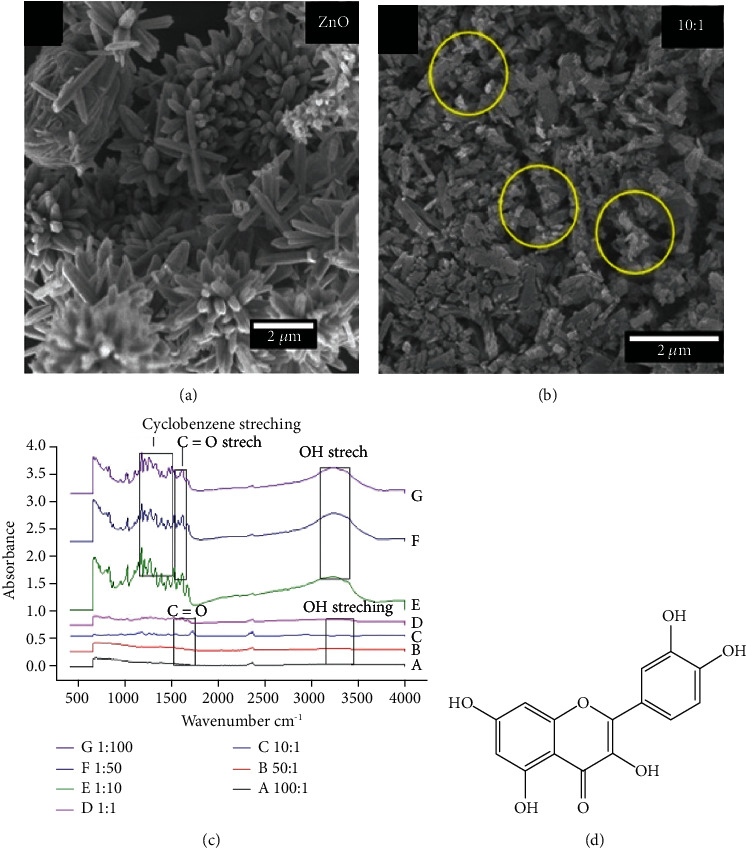
SEM images of (a) ZnO and (b) quercetin@ZnO nanoparticles (10 : 1). (c) FTIR results of quercetin@ZnO nanoparticles (ZnO : quercetin): a—100 : 1, b—50 : 1, c—10 : 1, d—1 : 1, e—1 : 10, f—1 : 50, and g—1 : 100. (d) Chemical structure of quercetin.

The characterization of the ZnO NPs and quercetin@ZnO is illustrated in Figure 1. SEM evaluated the physical appearance of ZnO NPs, images were taken on different magnifications, and the ZnO image was scanned at 2 *μ*m which showed a flower-like arrangement of ZnO NPs with clusters of flowers (Figure 1(a)). SEM imaging of quercetin@ZnO NPs was also scanned on different magnifications (Figure 1(b)), and different ratios of ZnO nanoparticles and quercetin@ZnO were prepared to select a suitable ratio (quercetin : ZnO, 1 : 10) for the experiments and clearly showed that quercetin is loaded on ZnO NPs encircled by yellow dotted lines. However, loading is prominent (Figure 1(b)). Moreover, quercetin : ZnO in the ratio of 1 : 50 has much lower loading than 1 : 10; similarly, the 1 : 100 ratio of quercetin : ZnO has too much low concentration of quercetin due to 100 percent concentration of ZnO NPs and only 1 percent concentration of quercetin (data not shown). Nevertheless, as quercetin's concentration increased in different quercetin : ZnO NPs, ZnO's morphologies get changed and were not prominent (data not shown).

#### 3.1.2. FTIR Analysis of ZnO and Quercetin@ZnO NPs

FTIR spectrums were recorded to examine the quercetin encapsulation with ZnO NPs (Figure 1(c)). Different concentrations of ZnO : quercetin NPs were taken, i.e., (a) 100 : 1, (b) 50 : 1, and (c) 10 : 1, which exhibited a peak at 1500 cm^−1^ to 1600 cm^−1^ due to C=O stretching and at 3200 cm^−1^ to 3500 cm^−1^ due to OH stretching. These peaks showed a high concentration of ZnO NPs and low quercetin concentration, but the peaks indicated both functional groups of ZnO and quercetin. Nevertheless, in ZnO : quercetin—(d) 1 : 1, the equal concentration of ZnO NPs and quercetin showed an absorbance peak at 1400 cm^−1^ to 1600 cm^−1^ due to cyclobenzene stretching in quercetin and a peak at 1500 cm^−1^ to 1600 cm^−1^ due to carbonyl in both quercetin and ZnO, while the peak at 3200 cm^−1^ to 3500 cm^−1^ was due to OH stretching (Figure 1(c)).

Similar peaks appeared when the concentration of quercetin was increased, i.e., ZnO : quercetin—(e) 1 : 10, (f) 1 : 50, and (g) 1 : 100, but with great intensity mainly because of the high concentration of quercetin (Figure 1(c)). These results showed successful encapsulation of quercetin with ZnO NPs to form quercetin@ZnO. The complete structure of quercetin showed multiple bonding sites (Figure 1(d)).

### 3.2. Drug Loading and Release

#### 3.2.1. Adsorption Rate (AR) and Drug Loading Capacity (LC) of ZnO NPs

**Figure 2:**
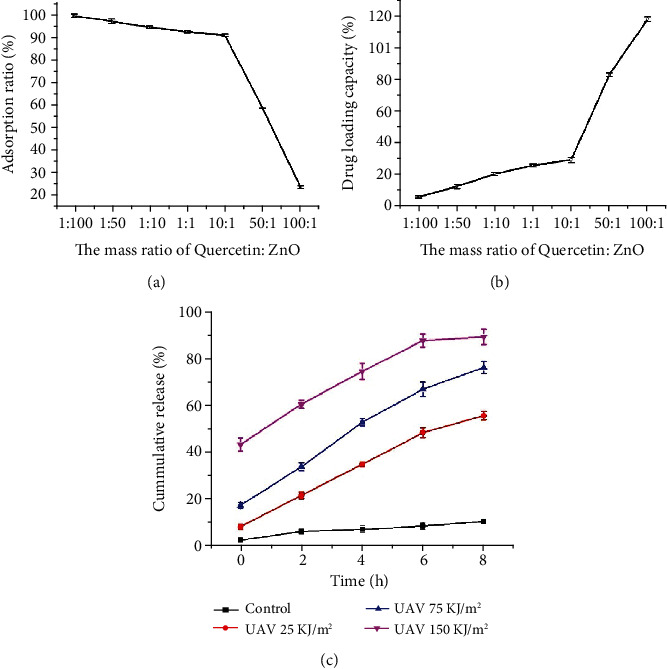
(a) The reliance on percent adsorption rate (AR) of quercetin on ZnO NPs on the mass ratio of quercetin to ZnO. (b) The reliance on loading capacity (LC) of quercetin on ZnO NPs on quercetin's mass ratio to ZnO. (c) Quercetin's UV-controlled release behaviour for various time points under control, 2, 4, 6, and 8 h following UVA exposure.

The reliance on such nanocarriers loaded with drug molecules is purely based on the AR and LC to mass ratio and herein was determined for both ZnO NPs to quercetin (Figures 2(a) and 2(b)). The highest AR could run up to 99.09 ± 0.69% (Figure 2(a)), when the mass ratio of quercetin : ZnO was 1 : 100; the LC was unsatisfactory (Figure 2(b)). Overall, the mass ratio of 10 : 1 (quercetin : ZnO) received an optimal AR of 90.61 ± 0.59% (Figure 2(a)) and LC of 29.35 ± 1.62% (Figure 2(b)). We were delighted that our analogously round and monodisperse ZnO NPs could get an optimal AR of 90.61 ± 0.59% and LC of 29.35 ± 1.62%; hence, maximum penetration capability can be achieved as previously reported [22, 24, 25].

### 3.3. UV-Controlled Drug Release Behaviour of Quercetin@ZnO

It is aimed at developing UV-controlled release of drugs which is the main concept of this article using quercetin@ZnO NPs to report a potential source for the antiphotoaging effect by adding an iron quenching antioxidant into the skincare formulations. Keeping this in view, concerning quercetin's strong iron chelation capability, this study was designed to evaluate the UV-driven release of the drug from ZnO NPs (Figure 2(c)). At 8 h of UVA exposure (150 kJ/m^2^), the collective release percent of quercetin was up to 88.71 ± 3.29%, while 25 kJ/m^2^ and 75 kJ/m^2^ UVA doses showed 55.29 ± 1.84% and 75.71 ± 2.60% quercetin release, respectively (Figure 2(c)). On the contrary, the quercetin@ZnO NPs were not exposed to UVA radiation, and only 10.29 ± 1.15% of quercetin was released at 8 h while keeping this in the dark (Figure 2(c)); hence, it proves that the UVA irradiation has some stimulating effects mainly because the UV light creates hydrophobic/hydrophilic transitions in ZnO NPs [22, 23].

### 3.4. Measurement of Redox Potential and Iron-Binding Capability of Quercetin

**Figure 3:**
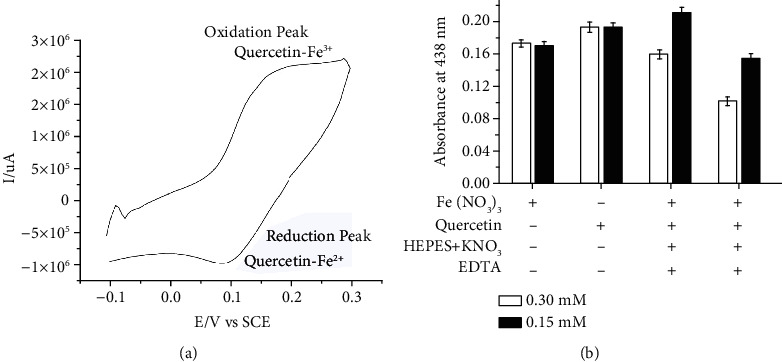
Cyclic voltammogram of quercetin-Fe complex (2 : 1) in phosphate buffer solution pH 7.0 at a scan rate of 0.1 V (a). Iron binding of the quercetin-Fe^3+^ complex with 0.15 mM and 0.30 mM of Fe(NO_3_)_3_ at 430 nm, which is the *ƛ*_max_ of the complex (b).

The cyclic voltammogram (CV) of the quercetin-Fe^3+^/quercetin-Fe^2+^ complex exhibited a reversible redox process with a cathodic peak potential EPc of 0.19 V and an anodic peak potential EPa of 0.09 V versus saturated calomel electrode (Figure 3(a)). From these two values, the E1/2 was calculated to be 0.14 V versus a saturated calomel electrode, which corresponds to 0.38 V versus NHE (normal hydrogen electrode). The iron-binding capability of quercetin was examined by spectrophotometry (Figure 3(b)). Here, we used different concentrations (0.15 mM, 0.30 mM) of iron nitrate (Fe(NO_3_)_3_) and treated it with quercetin. In the ligand exchange reaction, the absorbance decline at 438 nm showed an absolute elimination of Fe^3+^ by EDTA from the quercetin-Fe^3+^ complex (Figure 3(b)). It is a clear indication of absorbance due to the capturing of Fe^3+^ by quercetin at 0.15 mM iron nitrate (Figure 3(b)). These results showed that quercetin could reduce ROS and can prevent UV damage. Similar experiments for higher concentrations (0.30 mM) of Fe(NO_3_)_3_ were also performed (Figure 3(b)), which gave similar results and where quercetin showed decreased absorbance at 438 nm.

### 3.5. ROS and Anti-Inflammatory Potential

**Figure 4:**
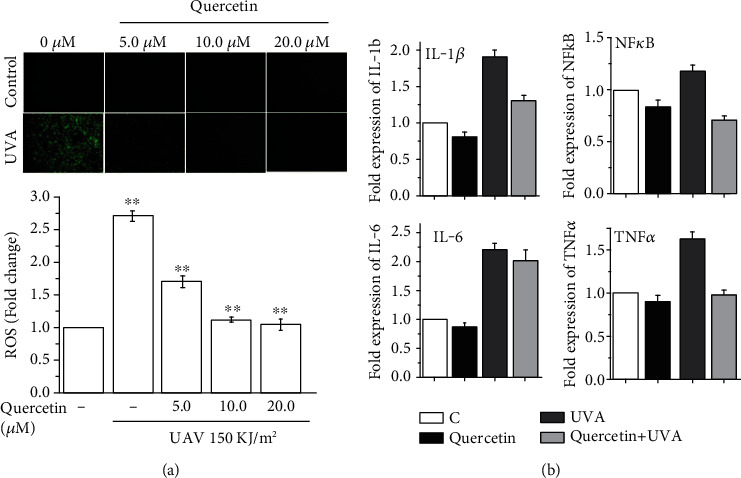
Quercetin@ZnO NPs modulate UVA-induced oxidative stress examined by the relative intensity of fluorescence (a) and inflammatory response (b) in HaCaT cells (^∗^*p* < 0.05, ^∗∗^*p* < 0.01).

Quercetin@ZnO NPs are not involved in the reflection or absorption of UVA by the ZnO; instead, they may dissipate the radioactive energies by releasing the quercetin from the ZnO molecules. UVA is known to create its damaging effects on the skin mainly by the generation of oxidative stress by ROS, which interacts with various intracellular components and specifically the lipid membranes. Quercetin is a natural phytochemical sourced in a considerable number of fruits and vegetables and is reported to reduce oxidative stress and diminish the damage incurred by ROS. In the current study, when the HaCaT cells were exposed to UVA (150 kJ/m^2^), which causes the generation of ROS that gave higher fluorescence when stained with dihydroethidium (Figure 4(a)), indicating the production of the high amount of intracellular ROS levels, and when the HaCaT cells were pretreated with various indicated quercetin concentrations (Figure 4(a)), the relative intensity of fluorescence caused by ROS was significantly reduced even up to normal sham cells (Figure 4(a)). The UVA-mediated ROS generation was lowered as pretreated quercetin's concentration gradually increased by neutralizing the ROS efflux in HaCaT cells (Figure 4(a)).

Reducing the expression of inflammatory cytokines may help reduce or slow down the photoaging process in skin cells. Herein, we examined major inflammatory factors' expression playing a decisive role in the onset of photoaging following UVA exposures (Figure 4(b)). The expression levels of various inflammatory factors, viz., IL-1*β*, IL-6, NF-*κ*B, and TNF-*α*, were also studied in HaCaT cells through qRT-PCR. Quercetin@ZnO NPs showed a significant reduction in UVA-mediated expression of the IL-1*β* level from 1.9-fold to 1.3-fold, reduce the level of IL-6 from 2.2-fold to 1.8-fold and the level of NF-*κ*B from 1.18-fold to 0.71-fold, and reduce the levels of TNF-*α* from 1.63-fold to 0.93-fold (Figure 4(b)). The reduction in the level of inflammatory factors indicated a strong effect of quercetin@ZnO NPs on reducing inflammations in HaCaT cells.

### 3.6. Cytotoxicity of Quercetin and Biocompatibility

**Figure 5:**
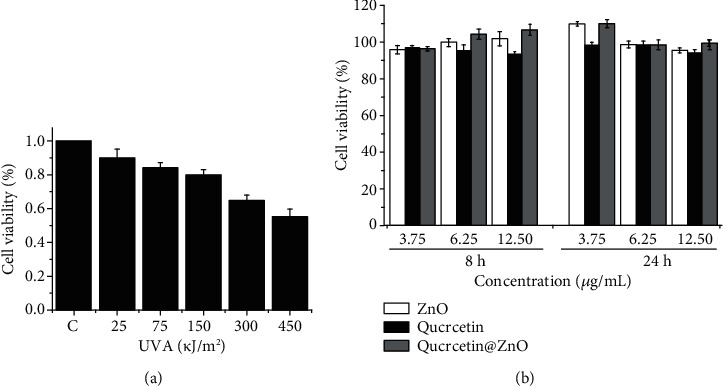
Quercetin's effect on UVA-induced cytotoxicity (a) in human keratinocytes HaCaT at 8 and 24 h (b).

The cytotoxicity assay assessed the use of quercetin@ZnO NPs against UVA for skin protection using HaCaT cells (Figure 5). Biocompatibility is the main criterion for utilizing biomaterials in clinical applications.  Figure 5(a) shows the cell viability loss upon exposure to various UVA doses, but approximately 80% cell viability was achieved at 150 kJ/m^2^, which is considered the optimum dose and used in all experiments herein and as reported in the literature [26]. Herein, increasing the UVA dose decreases the viable cells at 24 h of postirradiation (Figure 5(a)). The effect of quercetin@ZnO NPs on UVA-induced cytotoxicity in HaCaT cells was also checked at 8 and 24 h, and no cytotoxicity on the growth of HaCaT was seen (Figure 5(b)). Moreover, quercetin@ZnO NPs in various ratios and concentrations were applied to HaCaT cells, and all concentrations had no harmful effect and cells showed proliferative behaviour (Figure 5(b)).

## 4. Discussion

UVs contribute to photoaging which is sought mainly due to UVA-mediated iron release in the cells, which creates efflux of ROS, and through various cellular signalling cascade degradation of the ECM and wrinkled skin [17]. ROS creates cellular oxidative stress in human skin leading to multiple issues in skin tissue [8–10] by direct or indirect damage of the natural defense system of the skin which ultimately responds to these stressors [9, 12]. Quercetin is well known for its ROS-scavenging activities and is more active than many known antioxidants like vitamin E. Quercetin being the dietary flavonoid source in multiple foods (vegetables, fruits, tea, brewed drinks, etc.) has potential beneficial health effects mainly because of its potent antioxidant nature [19]. Besides ZnO, quercetin is also encapsulated with chitosan/alginate, where it has shown effective therapeutic potential against oxidative stress-induced liver injury [27].

Primarily, the current study was aimed at demonstrating the use of quercetin@ZnO NPs, designed for potential application as antiphotoaging mainly by quenching the UVA-mediated free cellular iron to provide cytoprotection at the same time. Human skin HaCaT cells play a major role in the progression of various skin diseases and particularly premature photoaging, and hence, the quercetin@ZnO NPs were applied on HaCaT cells to elucidate their therapeutic potential. Different studies reported the use of various antioxidants providing cytoprotection which have been encapsulated onto either the ZnO NPs or chitosan, which are well-known drug carriers [22, 28]. Using biocompatible materials liable to any particular physical incitement or following any stimuli which may undergo a hydrolytic cleavage, protonation or (supra) molecular conformational changes to release the attached drug are sought to deliver drug molecules at specified sites [29, 30]. UV light/dark-driven consecutive cycles produced an on-demand drug delivery system which is biologically compatible and efficient for shielding skin cells/tissue from UV light which was built up with ZnO [22, 23]. Moreover, ZnO NPs have got strong stability, biocompatibility, and cost-effectiveness but are widely used in drug delivery systems for long [31]. Multiple structural isomorphs have been reported in the literature such as mesoporous ZnO, which can uphold doxorubicin in nanoscale assemblies via adsorption and be released following a slight change in pH or ultrasound waves [32].

Iron is one of the frequent but essential trace elements in the human body, and both Fe^2+^ and Fe^3+^ are reported to take part in different essential physiological processes in almost all eukaryotic cells [33–35]. Due to its widespread nature, iron may quickly donate or accept electrons by engaging in single-electron exchange reactions and potentially producing or amplifying intracellular ROS [36]. These iron-generated ROS can damage cellular lipid layers, nucleic acids, and proteins [4]. Despite the essential roles of iron in the body, it can be toxic if it accumulated in excess and lead to various diseases including cancer, ischemia-reperfusion injury in transplantations, and aging-associated neurodegenerative issues [7]. Furthermore, free cellular iron may induce various types of cutaneous damage by disrupting cellular membranes to cause lipid peroxidation and denaturation of the extracellular matrix leading to premature photoaging [37]. Keeping in view the cutaneous damaging effects of free cellular iron, it is stressed to explore efficient iron sequesters and use skincare products to avoid or slow down the premature photoaging [37]. Quercetin is said to be a potent inhibitor of iron absorption and influence acute and chronic intestinal absorption of iron either by chelation, regulating ferroportin transporter expression inside the intestinal lumen using its 3-hydroxyl group, or by directly altering tissue iron distribution [38]. In the current study, quercetin@ZnO NPs were developed to sequester redox iron in order to avoid any interpersonal quercetin bioavailability issues. This may reduce premature photoaging caused by chronic exposures to solar UV-mediated iron overloads. This approach needs further in-depth study to explore the cell signalling cascades driven by quercetin's targeted release following UVA exposures.

Attempts have been made to use various drugs to encapsulate ZnO to cure various skin ailments, and these drugs use multiple cellular signalling pathways. Quercetin is a prominent flavonoid having antioxidant, anti-inflammatory, and anticancer actions all at once [39]. Quercetin helps reduce the inflammatory cytokine burst in dendritic cells (both myeloid and plasmacytoid dendritic cells) by modulating the transcriptional activation of activator protein 1 (AP-1), secretory leucoprotease inhibitor (Slpi), and heme oxygenase-1 (Hmox-1) genes [40]. Quercetin showed to be effective against UVB radiation-mediated damage by downregulation of COX-2 *in vitro* [41]. In another study, quercetin-loaded chitosan NPs were successfully prepared to reduce UVB-induced damage in HaCaT cells mainly by lowering the inflammatory responses by downregulation of the NF-*κ*B/COX-2 signalling pathway [42]. Herein, the current study showed that mimicking results for quercetin@ZnO NPs have strong antioxidant potential and reduce the expression of various inflammatory factors, viz., IL-1*β*, IL-6, NF-*κ*B, and TNF-*α*. Quercetin@ZnO seems to have the potential antitumor therapy [43] and hence could be focused to execute this novel approach of using Fe sequestration, cytoprotection, or apoptotic cell deaths.

*In vivo* application of quercetin is relatively low due to less hydrophilicity and little or no absorption at all by the cutaneous tissues [44]. Moreover, bioavailability of quercetin is mainly due to considerable variations among different individuals [45]. Hence, new but universal approaches are being made either directly or indirectly to deliver quercetin mainly using ZnO NPs at the targeted site. ZnO nanoparticles have shown superiority among related NPs in drug delivery systems. Herein, the UV reflection property of ZnO is utilized to lessen harmful effects of UVA as ZnO actively lodges much of the energy-loaded photons and some of them aid in the discharge or release of quercetin molecules correctly. Hence, further protection to the HaCaT cells even if extreme UVA exposures release ROS burst can be achieved.

## 5. Conclusion

The results led us to conclude that quercetin@ZnO NPs are entirely developed and characterized, showing that efficient quercetin release actions were found under controlled UVA exposure. Quercetin@ZnO NPs not only reflect UVA light but dissipate the energy-rich photons to release quercetin molecules that bear exceptional antioxidant, anti-inflammatory, and iron-sequestrating potential. Keeping in view the superior biocompatibility, quercetin@ZnO NPs can potentially be combined with UVA for simultaneous protection and medication for antiphotoaging therapeutics.

## Figures and Tables

**Table 1:** Primer sequences for qRT-PCR analyses.

No.	Gene name	5′-3′	Primer sequence 5′-3′
1	GAPDH	F	GAAGATGGTGATGGGATTTC
		R	GAAGGTGAAGGTCGGAGTC

2	IL-1*α*	F	TGAGCTCGCCAGTGAAATGA
		R	AACACGCAGGACAGGTACAG

3	IL-6	F	CTCAATATTAGAGTCTCAACCCCCA
		R	GAGAAGGCAACTGGACCGAA

4	NF-*κ*B	F	AACAGAGAGGATTTCGTTTCCG
		R	TTTGACCTGAGGGTAAGACTTCT

5	TNF-*α*	F	CTGGGCAGGTCTACTTTGGG
		R	CTGGAGGCCCCAGTTTGAAT

## Data Availability

The original data can be asked from the first and corresponding authors.

## Abbreviations

AR: Adsorption rate
cDNA: Complimentary DNA
COX-2: Cyclooxygenase 2
CV: Cyclic voltammetry
DCFH-DA: 2′,7′-Dichlorofluorescein diacetate
DMEM: Dulbecco's modified Eagle's medium
DNA: Deoxyribonucleic acid
ECM: Extracellular matrix
EDTA: Ethylenediaminetetraacetic acid
FBS: Fetal bovine serum
FeNO_3_: Ferric nitrate
FTIR: Fourier transform infrared spectroscopy
HaCaT: Human epidermal keratinocytes
IL-1*β*: Interleukin 1*β*
IL-6: Interleukin 6
kJ/m^2^: Kilojoule/square meter
KNO_3_: Potassium nitrate
LC: Loading capacity
LIP: Labile iron pool
mM: Millimolar
MMPs: Matrix metalloproteinase
NF-*κ*B: Nuclear factor kappa B
NPs: Nanoparticles
OD: Optical density
PBS: Phosphate buffer saline
qRT-PCR: Quantitative real time polymerase chain reaction
RNA: Ribonucleic acid
ROS: Reactive oxygen species
SD: Standard deviation
SEM: Scanning electron microscope
TNF-*α*: Tumor necrosis factor alpha
UVA: Ultraviolet radiation A
UVB: Ultraviolet radiation B
UVR: Ultraviolet radiations
ZnO: Zinc oxide
*μ*L: Microlitre.
